# Supplementary material for: Detection of Potato Pathogen Clavibacter sepedonicus by CRISPR/Cas13a Analysis of NASBA Amplicons
Source: Int J Mol Sci. 2024 Nov 14;25(22):12218. doi: 10.3390/ijms252212218 (PMC11595182; doi:10.3390/ijms252212218)
Supplement: Supplementary file 1 [file ijms-25-12218-s001.zip › ijms-3253118-supplementary.pdf]

Supplementary Materials

for

Detection of potato pathogen Clavibacter sepedonicus by CRISPR/Cas13a analysis of NASBA amplicons

Svetlana A. Khmeleva, Leonid K. Kurbatov, Konstantin G. Ptitsyn, K., Olga S. Timoshenko, Darya D. Morozova, Elena V. Suprun, Sergey P. Radko and Andrey V. Lisitsa

Table S1. Sequences of NASBA primers and DNA templates for the synthesis of gRNAs and the artificial RNA target. The sequence of T7 promoter is shown in italic; sequences coding gRNA spacers are underlined. In primers, the sequence complimentary to the targeted sequence is indicated in bold and the core sequence of T7 promoter – in italic.

Oligonucleotide name	Oligonucleotide sequence (5′ → 3′)
RNA target_template	accggaaacgtgcagagatgtgcgccccccaaggtggcta*ccctatagtgagtcgtatta*
gRNA1_template	ttgggggcgcacatctctgcacgtttctgttttagtccccttcatttttggggtggtcagtctaaatc*ccc tatagtgagtcgtatta*
gRNA2_template	ccttgggggcgcacatctctgcacgtttgttttagtccccttcatttttggggtggtcagtctaaatc*ccc tatagtgagtcgtatta*
T7F	*taatacgactcactataggg*
P2	cgatgcaacgcgaagaac
P1-205	*taatacgactcactatagg*g**ttggccccggcagtct**
P1-213	aattc*taatacgactcactataggg*a**ggttggccccggcagtct**
P1-215	aattc*taatacgactcactataggg*aga**ggttggccccggcagtct**
P1-218	aattc*taatacgactcactataggg*agaagg**ggttggccccggcagtct**
P1-222	aattc*taatacgactcactataggg*agaagg**ggttggccccggcagtctccta**

Table S2. Sequence (5′ → 3′) of the section of *C. sepedonicus* 16S rRNA gene, coding the section of 16S rRNA amplified by NASBA. Sequences corresponding to sites of primer annealing are underlined. Sequence corresponding to the 16S rRNA region recognized by the gRNA1 spacer is indicated in bold. Nucleotides different from those in other *Clavibacter* species are marked by red.

cgatgcaacgcgaagaaccttaccaaggcttgacatataccgga**aacgtgcagagatgtgcgccccccaagg**tcggtatacaggtggtgc atggttgtcgtcagctcgtgtcgtgagatgttgggttaagtcccgcaacgagcgcaaccctcgttctatgttgccagcacgtaatggtgggaa ctcataggagactgccggggccaacc

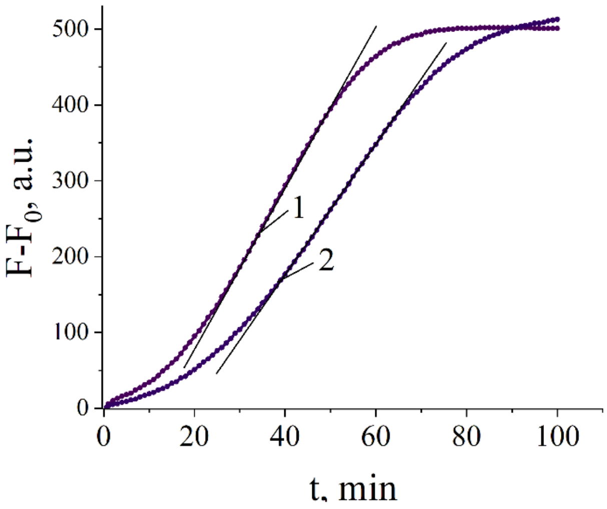

Figure S1. The differences between fluorescence (in arbitrary units, a.u.) of FQ reporters in the presence (F) and the absence (F_0) of the artificial RNA target (0.5 ng per reaction mixture) as a function of time. The concentrations of Cas13a and gRNA – 15 nM and 60 nM, respectively. The concentration of FQ reporters – 125 nM. Curves 1 and 2 correspond to gRNA1 and gRNA2, respectively. The linear segments of the curves are shown by black lines. The slopes of the lines (in a.u./min) were taken as V_0 values. The fluorescence measurements were carried out at 37°C on an Infinite M200 PRO plate reader (TECAN, Mannedorf, Switzerland).

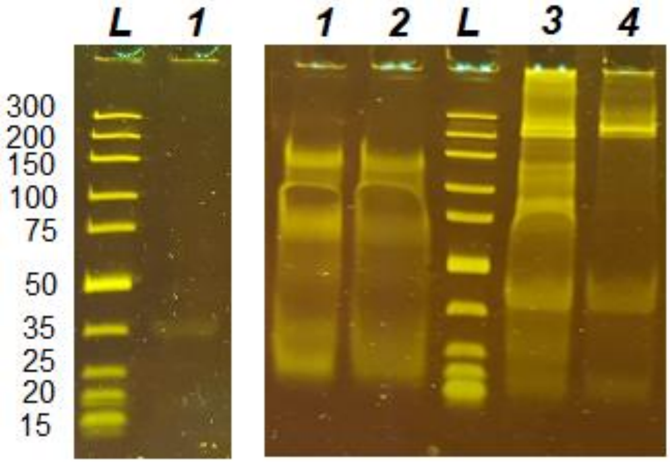

(a) **(b)**

Figure S2. The electrophoretic analysis of NASBA products for different variants of primer P1 (Table S1). (**a**): lane *1* – primer P1-205; lane *L* – DNA size standards (the size in base pairs is shown at the left). (**b**): lanes 1, 2, 3, and 4 – primers P1-213, P1-215, P1-218, and P1-222; lane *L* – DNA size standards. 10% polyacrylamide gel, TBE buffer (89 mM Tris-HCl, 89 mM boric acid, 2 mM EDTA, pH8.3), staining with SYBR Green fluorescent dye, visualization – with a SkyLight blue transilluminator (Vilber, Eberhardzell, Germany). NASBA was conducted with 10^5 copies of 16S rRNA per reaction.

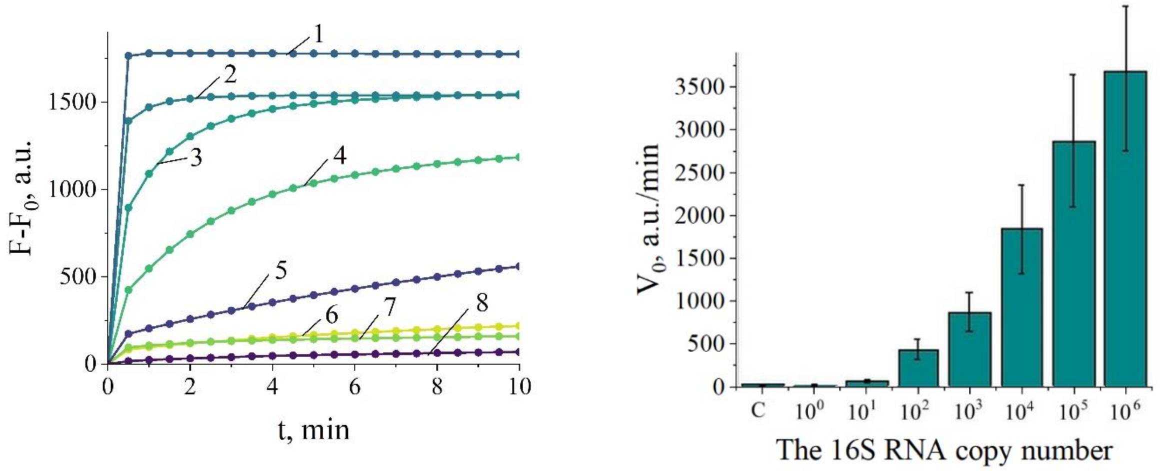

(a) (b)

Figure S3. The determination of *C. sepedonicus* 16S rRNA in the presence of 1 ng of potato total RNA with the NASBA/Cas13a detection system in the "two test tubes" format. (**a**): The representative curves of fluorescence kinetics, illustrating Cas13a analysis of NASBA products. F – fluorescence values measured in Cas13a analysis of NASBA samples, F_0 – those measured for the negative control (an aliquot of nuclease-free water was added instead of a NASBA sample). Curves 1, 2, 3, 4, 5, 6, and 7 correspond to NASBA carried out with the different number of 16S rRNA copies per reaction – 10^6, 10^5, 10^4, 10^3, 10^2, 10, and 1, respectively. Curve 8 corresponds to the Cas13a analysis of no template control for NASBA. (**b**): The values of the initial rate of fluorescence increase, V_0, in the Cas13a cleavage assay for the analysis of NASBA samples with different loads of RNA target per amplification reaction. "C" corresponds to the analysis of no template controls for NASBA. The mean values and confidence intervals for the confidence level p = 0.95 are shown.

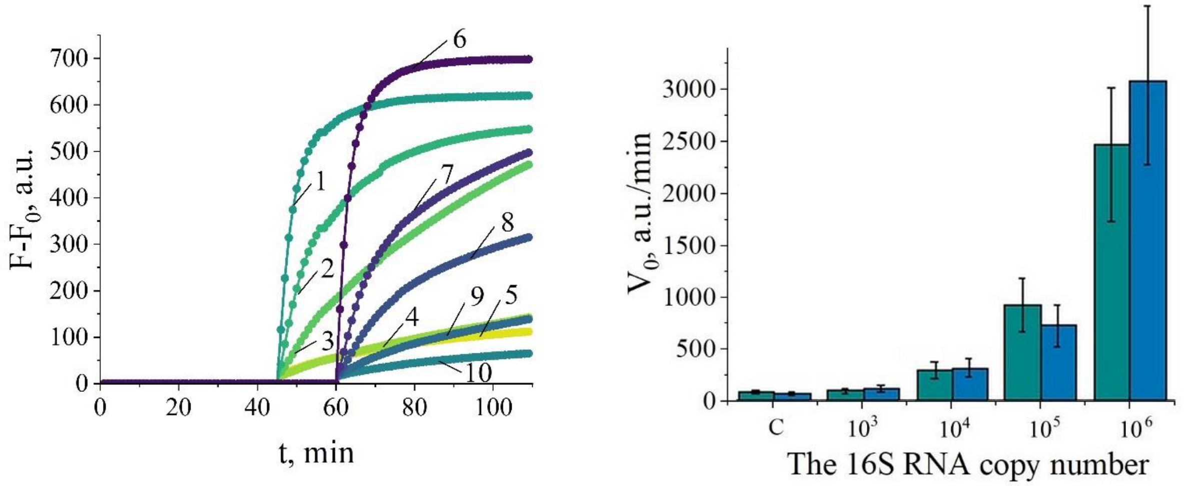

(a) (b)

Figure S4. The performance of the NASBA/Cas13a detection system in the "one-pot" format. (**a**): The representative curves of fluorescence kinetics. F_0 – the fluorescence intensity at the start of measurements. Curves 1, 2, 3, and 4 – loads of 10^6, 10^5, 10^4, and 10^3 copies of *C. sepedonicus* 16S rRNA per reaction and NASBA conducted for 45 min prior to mixing the 10-µL NASBA reaction with the 10-µL Cas13a cleavage assay mixture by a short spin of the reaction tube. Curves 6, 7, 8, and 9 – the same order of loads as above but NASBA conducted for 60 min. Curves 5 and 10 correspond to NASBA without the load of target (no template controls). (**b**): The V_0 values for the different loads of RNA target per reaction. Green and blue bars – 45- and 60-min NASBA, respectively. "C" – no template controls. The mean values and confidence intervals for the confidence level p = 0.95 are shown.

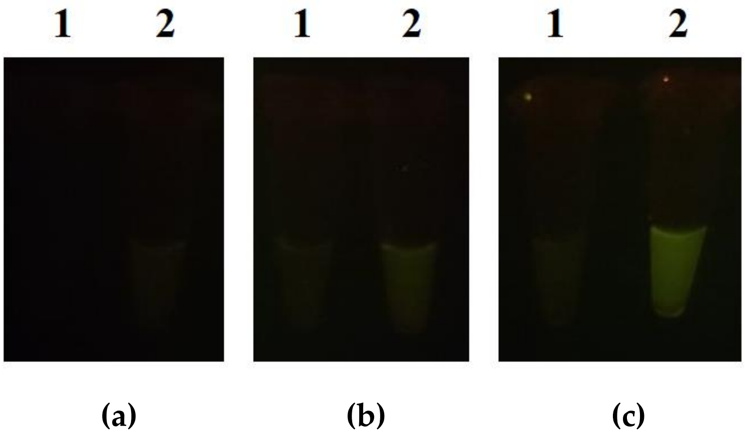

Figure S5. The optimization of FQ reporter concentration in the Cas13a reaction mixture for the *C. sepedonicus* determination in the "one-pot" format with the "naked-eye" detection. 1 and 2 – no template control and load of 10^4 copies of 16S rRNA per a NASBA/Cas13a reaction, respectively. Panels (a), (b), and (c) – FQ reporter concentrations of 0.125, 0.3, and 0,75 µM in the NASBA/Cas13a reaction mixture, respectively.

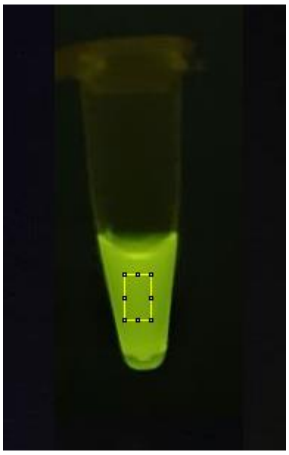

Figure S6. The illustrative example of a rectangular area inside the image of reaction mixture chosen to determine the density of fluorescence intensity.
